# Electron Microscopy of In-Plaque Phage T3 Assembly: Proposed Analogs of Neurodegenerative Disease Triggers

**DOI:** 10.3390/ph13010018

**Published:** 2020-01-18

**Authors:** Philip Serwer, Barbara Hunter, Elena T. Wright

**Affiliations:** 1 Department of Biochemistry and Structural Biology, The University of Texas Health Science Center, San Antonio, TX 78229–3900, USA; wrighte@uthscsa.edu; 2 Department of Pathology, The University of Texas Health Science Center, San Antonio, TX 78229–3900, USA; hunterb@uthscsa.edu

**Keywords:** assembly-generated particles, obscure, phage capsids, protein staining, protein structure, thin sections

## Abstract

Increased knowledge of virus assembly-generated particles is needed for understanding both virus assembly and host responses to virus infection. Here, we use a phage T3 model and perform electron microscopy (EM) of thin sections (EM-TS) of gel-supported T3 plaques formed at 30 °C. After uranyl acetate/lead staining, we observe intracellular black particles, some with a difficult-to-see capsid. Some black particles (called LBPs) are larger than phage particles. The LBP frequency is increased by including proflavine, a DNA packaging inhibitor, in the growth medium and increasing plaque-forming temperature to 37 °C. Acidic phosphotungstate-precipitate (A-PTA) staining causes LBP substitution by black rings (BRs) that have the size and shape expected of hyper-expanded capsid containers for LBP DNA. BRs are less frequent in liquid cultures, suggesting that hyper-expanded capsids evolved primarily for in-gel (e.g., in-biofilm) propagation. BR-specific A-PTA staining and other observations are explained by α-sheet intense structure of the major subunit of hyper-expanded capsids. We hypothesize that herpes virus triggering of neurodegenerative disease occurs via in-gel propagation-promoted (1) generation of α-sheet intense viral capsids and, in response, (2) host production of α-sheet intense, capsid-interactive, innate immunity amyloid protein that becomes toxic. We propose developing viruses that are therapeutic via detoxifying interaction with this innate immunity protein.

## 1. Introduction

**Figure 1:**
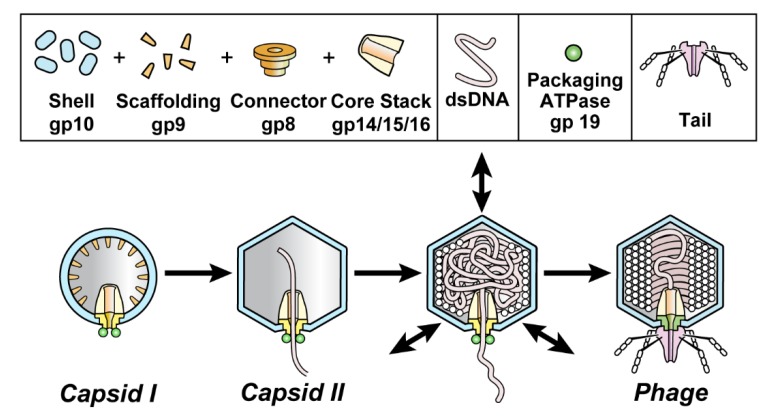
DNA packaging of the related phages, T3 and T7. Capsid I (left image) is assembled and then starts to package DNA while converting to capsid II (next image) and ultimately becomes a phage particle capsid (right image). The particles investigated here are dynamic capsid II expansion products that potentially mature to become capsids of phages; arrows in the third image from the left indicate expansion/contraction dynamism. Although pictured here as intermediates, intermediate status not been biochemically demonstrated for particles generated by shell dynamism. A detailed hypothesis is in reference 12.

An understanding of in vivo DNA transitions can be thwarted by obscurity of some transition products. Here, we use DNA packaging of phage T3 as a model and extend our previous work on de-obscuring products of DNA packaging. We do this by introducing electron microscopy (EM) of thin sections (EM-TS) of T3 plaques. DNA packaging for all studied double-stranded DNA phages is found to include assembly of a procapsid that subsequently packages DNA. Found necessary for packaging are a DNA packaging ATPase/endonuclease (gp19 for T3/T7; Figure 1; often called terminase) attached to a connector (gp8 for T3/T7; Figure 1; also called portal) [1,2,3,4,5]. T3/T7 proteins are named by gp (gene product), followed by the number [6] of the protein’s gene.

**Figure 2:**
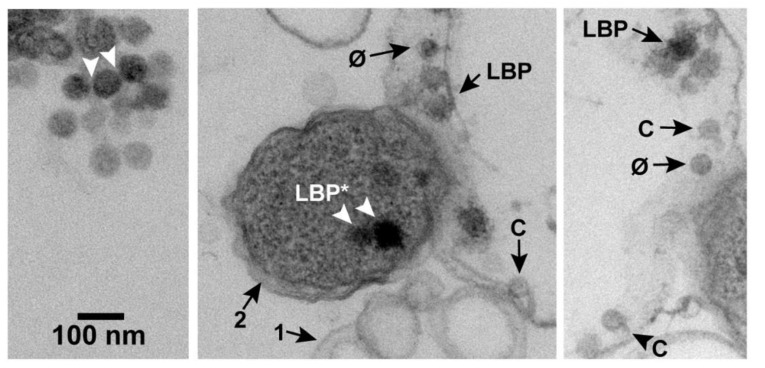
An image obtained by EM-TS of a phage T3 plaque. Annotations are described in the text. Uranyl acetate/lead staining was used. C, capsid; φ, phage; LBP, larger-than-phage black particle.

Part of the significance of T3 DNA packaging is that phage infections are relatively accessible models for pathogenic virus infections. For example, herpes viruses use the same packaging pathway. In addition, three DNA packaging-focused proteins of herpes viruses are homologs of the phage proteins with the same functions. These proteins are (1) the major protein of the capsid’s shell [7] (gp10 for T3/T7; Figure 1), (2) the portal protein [8,9] and (3) the terminase [10,11]. Indeed, the herpes virus portal was discovered via its phage homologs [8].

During assembly of the related phages, T3 and T7, the procapsid (called capsid I) undergoes a packaging-associated increase in size, increase in stability, increase in angularity and decrease in magnitude of (negative) average surface electrical charge density, σ. The result is a mature phage-like capsid (capsid II) (Figure 1; reviews [1,4]).

Within the last three years, lysate fractionation revealed products of T3 DNA packaging that were previously obscure. These products included particles (called ipDNA-capsids) with incompletely packaged DNA (ipDNA) packaged in capsids. Some ipDNA-capsids were larger and some smaller than phage [12,13]. This size variation was possibly generated post-lysis; i.e., the capsids might have been phage-sized before release from cells. The need to test for intracellular size-altered capsids was the initial stimulus for the current study. Analyzing capsids pre-lysis is best done by EM-TS of infected cells. Here, we find intracellular hyper-expanded, DNA-containing capsids by EM-TS of T3 plaques.

In-plaque (in-gel) analysis is used because (1) in-gel phage propagation occurs in the wild [14,15] and (2) in-gel propagation results in a gel fiber-induced increase in pressure on the T3 host [16]. The increased pressure may alter both cytoplasm and DNA packaging-generated particles, as discussed below. In-plaque, EM-TS analysis (1) is designed to qualitatively increase our knowledge of particles present and (2) cannot be as quantitative as studies done in liquid culture, which is presumably a reason that in-plaque, EM-TS analysis of DNA packaging has not previously been reported. We find that in-gel propagation increases the amount of intracellular, hyper-expanded capsids produced. We use these observations and analogies with herpes viruses to extend a general picture of the cause and therapy of neurodegenerative diseases.

## 2. Results

### 2.1. Large Particles

After plaque formation at 30 °C, some regions of each EM-TS-observed specimen had products of phage-induced bacterial damage. These products included (1) cells with partially leaked cytoplasm and (2) vesicles sub-cellular in size. Apparently, lysis-generated membrane fragments typically re-sealed to form these vesicles. A collection of these vesicles is in the middle panel of Figure 2 (arrow 1). A cell was assumed phage infected if it was a neighbor of these products of phage-induced damage. The three panels of Figure 2 were from a single micrograph from which two segments were removed, post reproduction, to enable an increase in the final magnification. The cytoplasm-deficient cell at the right of the center panel is the same as the cell in the right panel. The un-segmented, original micrograph is in Figure S1 of the Supplementary Materials. Most cells observed in-plaque were in clusters, as previously observed [16] for uninfected cells (not shown).

In the field of Figure 2, larger-than-phage, black particles (LBPs) were also present. An extracellular aggregate of LBPs is at the top of the left panel of Figure 2. The LBPs of this aggregate had the appearance of condensed, intracellular phage DNAs, previously seen for phages lambda and T4 [17,18,19,20,21]. In theory, the aggregated, extracellular LBPs also could have been precipitates of stain.

However, in practice, low-density space between adjacent, extracellular LBPs (arrowheads in the left panel of Figure 2) suggested presence of a capsid’s shell. This space was approximately 3 nm wide and was possibly occupied by two gp10 shells. A shell-like region was not seen on most of the remaining LBP surface, possibly because of shell thinness and low contrast. The diameter of the black region was 59–68 nm. The particle diameter was 62–71 nm, including the presumed shell. This diameter was larger than the diameter of phage particles and conventional capsid II. The diameters of the latter are investigated in Section 2.2. Further tests for an LBP-associated outer shell are in Section 2.4 and Section 2.6.

### 2.2. Phage-Sized Particles

In the absence of sectioning-induced distortion, the EM-TS diameter of a T3 capsid was expected to be the diameter in aqueous solution reduced by dehydration-caused shrinkage. If a particle’s EM-TS image had a visible outer shell, the section included an equator and observed diameter was particle diameter [21]. The average diameters of T3 phage, capsid II and ipDNA-capsid II were previously found by cryo-EM to be 60 nm [22]. However, dehydration has been found to reduce phage diameters by 12% (phage lambda [21]) to 22% (phage T7 [23]). Thus, the dehydration used in preparation for EM-TS was expected to reduce EM-TS diameters to 47–53 nm for T3 phage, conventional capsid II and ipDNA-capsid II. Some variability was expected because of the polygonal shape.

**Figure 3:**
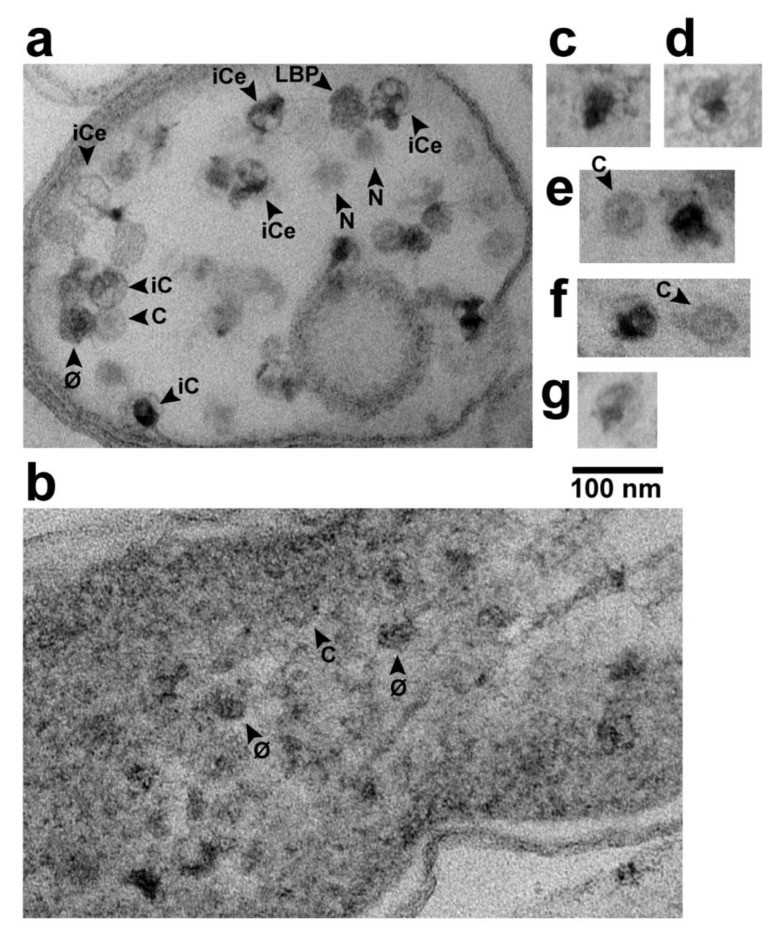
Phage assembly-generated particles seen in different contexts by in-plaque EM-TS. (**a**) the interior of a sub-cellular vesicle generated by cellular lysis and loss of cytoplasmic content, (**b**) the interior of an infected cell that has retained its cytoplasm with an end of the DNA-containing nucleoid at the upper right corner, (**c**–**g**) selected capsids with a hyper-expanded shell. Uranyl acetate/lead staining was used. φ, phage; C, capsid; iC, capsid with internal DNA; iCe, capsid with internal and external DNA; LBP, larger-than-phage black particle; N, LBP or φ, with equator not in the section.

Particles of expected phage size were seen inside of either lysis-generated, less than cell-size vesicles (Figure 3a) or apparent cells with mostly leaked cytoplasm (Figure 2, middle and right panels). In Figure 2, middle and right panels, we could not always discriminate rigorously between capsids and ipDNA-capsids. These particles have all been indicated by “C”. Particles more like phages (but, possibly ipDNA-capsids) have been indicated by “φ”. Both C and φ particles were 47–53 nm in diameter. They were significantly smaller than LBPs, as can be seen by visual comparison with the LBPs in Figure 2.

When C particles were in reduced-size vesicles (e.g., Figure 3a), capsid-associated DNA was more distinct than in Figure 2. Some C particles had ipDNA (particles labeled iC in Figure 3a). Some iC particles had visible external DNA (particles labeled iCe in Figure 3a), as though these particles were undergoing DNA packaging that had been interrupted by fixation. Both external and internal DNA were usually condensed to form a globule that, when internal, sometimes had branches (e.g., iCe particle at upper right in Figure 3a). The branches suggested that the packaged DNA had folds (kinks), as previously suggested by both data [24] and computer simulation [25]. Although C particles were best seen in cells and sub-cellular vesicles that were cytoplasm-deficient, both φ and C particles were sometimes seen, although less distinctly, while in cells with cytoplasm not detectably leaked (Figure 3b).

### 2.3. Intracellular LBPs

Additional, usually darker LBPs were observed in cells with full-appearing cytoplasm (e.g., the cell indicated by arrow 2, middle panel of Figure 2); these particles will be labeled LBP*. The average number per infected cell, *N*(LBP*), was 0.13 ± 0.06 in the specimen of Figure 2 (the Poisson sampling error is used here and below). The maximum number of LBP*s seen in a single cell was 2, after observing 500 infected cells. Images of LBP*s did not have perimeters as smooth as images of (extracellular) LBPs. At least part of the reason was cytoplasmic granularity. Some LBP* images were 4%–7% larger than images of the extracellular LBPs, e.g., the LBP* at the right in the middle panel.

Images of others were smaller, e.g., the LBP* at the left in the middle panel of Figure 2. The smaller size can be caused by absence of an LBP equator in the section. One LBP was observed in Figure 3a, along with images with the expected appearance of either an LBP or φ sectioned without an equator, e.g., particles labeled N in Figure 3a.

Some LBPs were also seen in cells with partially leaked cytoplasm (Figure 2, middle and right panels) and vesicles (Figure 3a). These LBPs also had the rough perimeter of the LBP*s, which is apparently the result of cytoplasmic adherence to these LBPs. A shell was not observed to surround the black region of these LBPs.

To further test for LBP* shells, we used two strategies. First, we systematically searched for these shells in the above specimens. Second, we (1) increased the *N*(LBP*) and (2) used a post-sectioning stain conjectured to be specific for shells of hyper-expanded ipDNA-capsids.

### 2.4. Strategy 1: LBP* Shells in Specimens of the Previous Section

The first strategy was pursued in the following way. Low-magnification (2000–10,000×) scanning of specimens was used to reveal cells that had partially leaked cytoplasm. Careful examination of each of these cells at higher magnification (20,000–40,000×) revealed that some had LBP*s. Images of these cells were then recorded at yet higher magnification, 60,000–120,000×.

**Figure 4:**
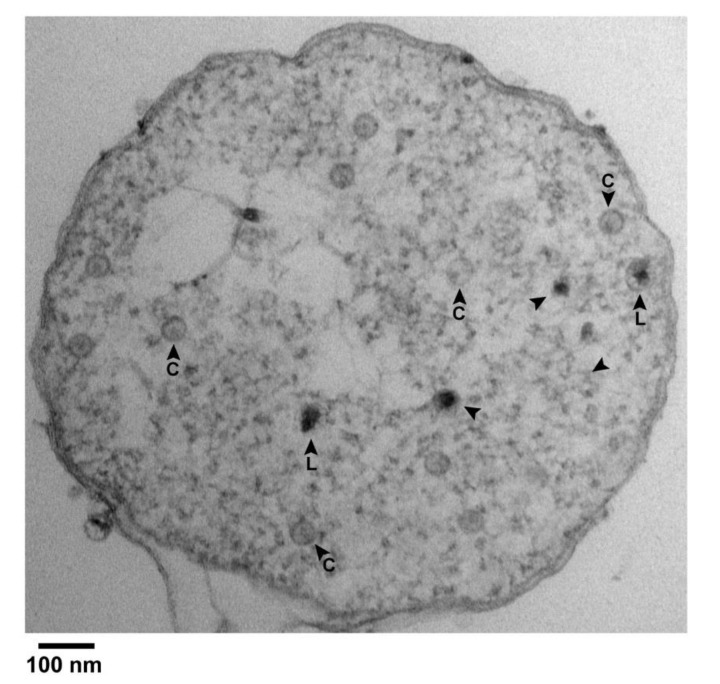
Black particles in a cell with partially leaked cytoplasm. Uranyl acetate/lead staining was used. An image is shown of the entire cross section of an infected cell. Arrowhead, black particle; C, capsid; L, large.

**Figure 5:**
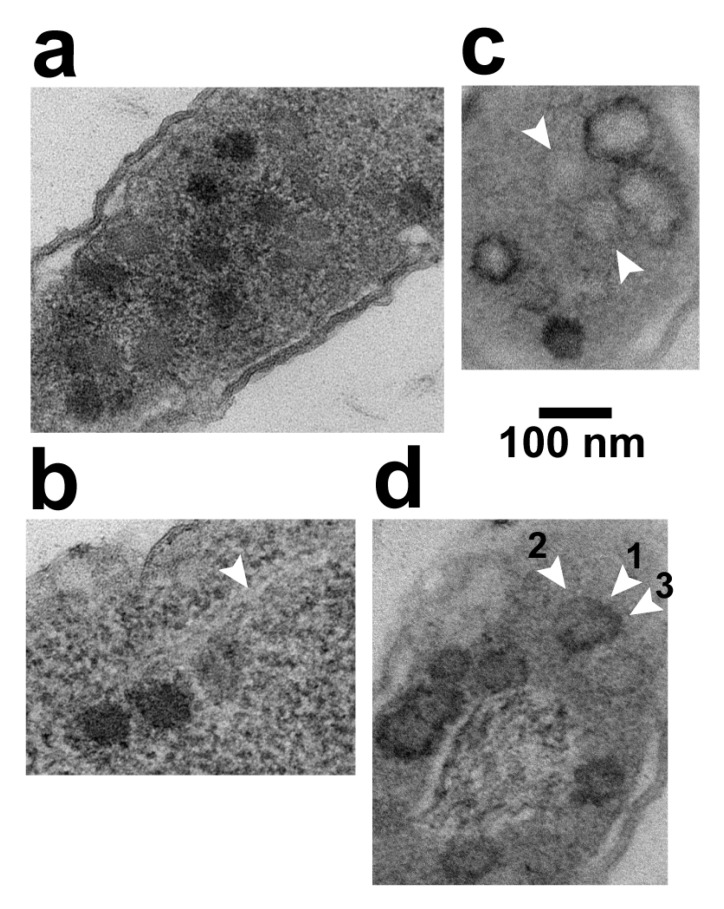
LBP*s and their shells. (**a**), (**b**) LBP*s in cells stained with uranyl acetate/lead; (**c**) as in (**a**,**b**) with A-PTA staining substituted for staining with lead/uranyl acetate; (**d**) as in (**a**), (**b**) with A-PTA staining after staining with lead/uranyl acetate.

A result was the finding of black particles that did have a shell surrounding most of the black (DNA) region of the particle. Figure 4 has several C particles (some labeled in Figure 4) and 5 black particles (arrowheads) in a cross-sectional image of such a cell. Of the black particles, two (arrowheads labeled L) had a shell larger than the shells of all C particles. The others may also have had such a shell, but the shell was not visible, possibly because sectioning did not encompass an equator. In any case, some segments of the L shells were so thin that they were borderline in visibility.

Higher magnification images of the two L particles are shown in Figure 3c,d. Other, similar particles were seen in other cells and vesicles with leaked cytoplasm. Of these, 3 are in Figure 3e–g; if a C particle is in the same field, the C particle is labeled. The average diameter of the L shells was 62 ± 2 nm. The black region was not always larger than the DNA-containing region of phage particles.

### 2.5. Strategy#2: A Proflavine-Induced Increase in N (LBP*) (Visualization of Single DNA Duplexes)

In the first step of our second strategy, we used a known specific inhibitor of T3 DNA packaging in an attempt to increase *N*(LBP*). Any increase would improve our capacity for analysis of possible LBP* outer shells. It would also improve the prospect for observing the conformation of unpackaged DNA segments near LBP*s.

The inhibitor was proflavine, which we used as an additive during T3 plaque formation. Proflavine had previously been found to specifically inhibit DNA packaging in liquid culture [26,27]. Initially, we found that 20 μg/mL proflavine (1) stopped T3 plaque formation at 30 °C, and (2) allowed plaque formation at 37 °C, although presumably with some inhibition of DNA packaging.

EM-TS of proflavine-altered plaques incubated at 37 °C revealed an increase in LBP* production. *N*(LBP*) underwent an increase to 3.9 ± 0.5. from 0.13 ± 0.04 without proflavine at 30 °C. This difference occurred even though lysis products were less frequent and, thus, the denominator of *N*(LBP*) may have been too high and *N*(LBP*) too low in the presence of proflavine. The maximum number of LBP*s in a single cell was 22, compared to two without proflavine. An image of LBP*s in one of the more LBP*-rich cells is in Figure 5a. In the absence of proflavine, incubation at 37 °C produced an *N*(LBP*) of 0.32 ± 0.04. Thus, proflavine had a minor assist from the elevated temperature in producing the increase in LBP* production observed.

The increased *N*(LBP*) assisted screening for the unusual circumstance in which cytoplasmic DNA molecules were visible while in apparently undepleted cytoplasm. In contrast to the collapsed unpackaged and packaged DNA when cytoplasm was depleted (Figure 3a), rare images were obtained with extended, DNA-like, cytoplasmic filaments. The infected cell in Figure 5b has 3 LBP*s at the lower left, with 2–3 extended filaments emanating to the upper right. In some regions (e.g., arrowhead in Figure 5b), the basic filament is seen to have a 2.0–3.0 nm width, approximately the 2.0 nm width of double-stranded DNA. The filaments were made visible, although at low contrast, by a surrounding cytoplasm that was slightly less electron dense than a filament. The filaments were not embedded in the more extensive, low electron density nucleoid, previously shown [17,18,19,20] to be where the bacterial chromosome resides (see also the discussion of Figure 5d, below). 

These observations suggested that the DNA molecule of at least some LBP*s was partially outside of the LBP and extended in comparison to DNA previously seen in Figure 3a. The extended DNA conformation was presumably caused by presence of cytoplasm. To our knowledge, this cytoplasmic DNA-extension phenomenon has not previously been reported.

### 2.6. Strategy #2: Alternative Staining

We then altered the staining procedure used for thin sections of proflavine-incubated, at 37 °C, T3 plaques. We either substituted acidic phosphotungstate (A-PTA; Methods Section) staining for, or added A-PTA staining to the traditional staining by lead and uranyl acetate. We used A-PTA staining because (1) some previous data suggested that hyper-expanded T3 capsids had gp10 subunits with α-sheet structure [13], (2) A-PTA staining had been found to preferentially stain compounds, including peptides, with high amino group density [28] and (3) α-amino groups are all on one edge of an α-sheet, where they generate a relatively high amino group density [29,30,31] and sometimes participate in phosphate and ATP binding/wrapping [30]. This is not the case for β-sheet [30,31]. We conjectured that phosphotungstate, like phosphate, would selectively bind to α-sheet and initiate a precipitate.

The result of either substituting or adding A-PTA staining was the finding that the LBPs became a minor feature in comparison to a not-previously-observed feature, black rings (BRs). BRs are illustrated in Figure 5c (substituting A-PTA staining) and 5d (adding A-PTA staining). Quantitatively, after adding A-PTA staining, only 1 LBP was seen while observing 50 BRs. *N*(BR) = 0.51 ± 0.07.

The BRs had a shape and size appropriate for PTA-precipitate formed on LBP-enclosing, icosahedral gp10 shells. They were 65–95 nm in diameter and were sometimes either pentagonal or hexagonal (e.g., Figure 5c). Nonetheless, some BRs had odd shapes. For example, in Figure 5c, the BR just below the top BR appeared to have a (lower) segment broken away from a polygonal segment. Others were missing most or all of the ring, but had a low-density center, as seen between the two arrowheads in Figure 5c. The BRs typically had an interior less dense than exterior when A-PTA staining was substituted (i.e., Figure 5c). The opposite was found when A-PTA staining was added (Figure 5d).

In uninfected cells, BRs were not a significant feature after A-PTA staining. Only two possibilities were observed in ~1600 cells randomly selected from two different specimens. These results indicate that the BR-forming particles were phage-produced. The only phage product of this size is a capsid.

The granularity of the surrounding cytoplasm was reduced by A-PTA staining. But, the surrounding cytoplasm was still dense enough to differentiate it from the bacterial nucleoid. In Figure 5d, a nucleoid, with DNA, is surrounded by several BRs.

The following observation confirmed the conclusion that BRs were formed by staining of hyper-expanded capsid shells. Occasionally, part of the BR-associated A-PTA precipitate pulled away (arrowhead 1 in Figure 5d) from a weakly contrasted, hyper-expanded shell (arrowhead 2 in Figure 5d), while the remainder of A-PTA-precipitate (arrowhead 3 in Figure 5d) remained in place. Just below this partial BR was a more weakly contrasted, hyper-expanded, 76–81 nm-diameter gp10 shell that had, around its entire perimeter, insufficient PTA to be called a BR. Mobility of PTA precipitate has been previously shown [28].

### 2.7. Results for Liquid Culture

*N*(BR) in liquid, 30 °C, broth culture was determined after termination of infection (multiplicity of infection = 5) at 18 and 22 min. Both termination times were in the DNA packaging window [26,27]. All cells were assumed infected. Membrane-containing lysis products were neither expected nor observed. The cells were not clustered as they were in-plaque. If BR production is the same in-plaque as in-liquid, then *N*(BR) should be higher in-liquid because the in-plaque data sample the entire T3 life cycle.

However, the opposite was found to be the case. The in-liquid *N*(BR) was 0.010 ± 0.003 at 18 min and 0.021 ± 0.006 at 22 min. These numbers are significantly lower than 0.98 ± 0.08 found in-plaque. When 20 μg/mL proflavine was added at 13.0 min after infection of the liquid culture, the values of *N*(BR) were also significantly lower in-liquid than in-plaque: 0.046 ± 0.01 in-liquid at 18.0 min and 0.006 ± 0.003 in-liquid at 22 min. This was in contrast to 0.51 ± 0.07 in-plaque.

## 3. Discussion

### 3.1. Intracellular Hyper-Expanded Capsids: Proposed Conformation for gp10

Probing for and characterizing of new, DNA packaging-generated particles is essential to improving the accuracy of hypotheses, as discussed below. Nonetheless, the particles observed here are not entirely new. Intracellular, hyper-expanded ipDNA-capsids of phage lambda were detected, although not characterized, over 40 years ago by EM-TS of cells infected in liquid broth culture [17]. Extended α-sheet structure was not considered and was, at the time, not well known, although it had already been reported (in 1951 [29]).

In addition, as the procapsid concept was being developed in the 1960s, some intracellular phage T4 capsids were found by EM-TS to have extra space between the outer capsid shell and the packaged DNA [18]. This space was a probable sign of hyper-expansion. These T4 capsids were difficult to observe [18]. Finally, neither the lambda nor the T4 capsids in these EM-TS detected states were isolated, although attempts were reported for T4 [18].

In contrast, isolation of capsids [32] and ipDNA-capsids [12,13] with hyper-expanded gp10 shells has been achieved for T3. In support of a role for shell dynamics in DNA packaging, evidence has been obtained for capsid shell-associated ATPase activity (T4 [33] and T3 [34]). Finally, ATP-induced contraction of purified, hyper-expanded ipDNA-capsids (T3 [12]) has been observed.

We suggest predominant α-sheet structure for BR-associated gp10. The reason is that this structure explains four different characteristics of hyper-expanded T3 ipDNA-capsids: (1) ATP-responsiveness [12], because bent α-sheet is an ATP-binding conformation [30], (2) negative and relatively high magnitude of σ, which is expected if the gp10 alpha sheet has its α-carboxyl edge on the capsid’s exterior [13], (3) EM-observed apparent staggering of gp10 shell subunits, which would minimize electrostatic repulsion of neighboring α-sheet-gp10 subunits [13] and (4) A-PTA staining (above).

### 3.2. Phage DNA Packaging

The data obtained here imply that at least some of the previously isolated hyper-expanded capsids [32] and ipDNA-capsids [12] were hyper-expanded in vivo, i.e., they were hyper-expanded before cellular lysis. For in vitro investigation of pathways, in the future, isolation/characterization analysis should be performed with lysates of cells infected in-gel. EM-TS should be used to check isolation/characterization analysis for completeness, as done during discovery of organelles of eukaryotic cells [35,36].

The following observations provide possible explanation for our finding of an in-gel increase in capsid hyper-expansion. Elevated pressure on the cell surface biases bacterial cytoplasm toward colloidal glass-like states [37,38], even at less than 0.7 atmospheres [38]. In typical media, *E. coli* has a higher internal osmotic pressure, 3–10 atmospheres, depending on conditions [39,40]. When *E. coli* elongates in-agarose gel, this internal pressure pushes aside gel fibers [16]. Reactive force on cells (Newton’s Second Law) is likely at least 0.7 atmospheres, sufficient to glassify cytoplasm.

Thus, the hypothesis is that cytoplasm glassified by in-gel propagation favors capsid hyper-expansion-driven T3 packaging. In the wild, in-gel propagation occurs in biofilms. Thus, we also hypothesize that hyper-expanded capsid states evolved primarily for DNA packaging during in-biofilm infections. A hyper-expansion-driven DNA packaging mechanism has been proposed [12].

### 3.3. Potential Relationship to Neurodegenerative Disease Causation

For neurodegenerative diseases, hypotheses are needed to explain and integrate the following observations: (1) correlation of previous herpes virus infection with subsequent Alzheimer disease [41,42], (2) correlation of Alzheimer disease and other neurodegenerative diseases with the presence of amyloid protein in a toxic conformation [43,44] and (3) correlation of α-sheet conformation with amyloid protein toxicity [45]. To explain and integrate these observations, one hypothesis is that some innate immunity systems recognize viral infections by recognizing α-sheet conformation of immature viral assemblies. These systems counter virus infection by production of innate immunity protein that adopts α-sheet conformation and, then, binds to and extends viral α-sheet and, thereby, blocks virus production [13]. The α-sheet-conformer of innate immunity protein is toxic, possibly because of capacity [30] to create membrane channels. It is detoxified via α-sheet to β-sheet conversion, β-sheet being the dominant conformation observed (review [13,43,44,46,47]). Disease is caused by hyper-activity of this innate immunity [46,47], possibly via mistaking of innate immune α-sheet as viral [13].

Phage/herpes virus assembly homologies (Introduction) support considering the possibility that, as seen here for the production of T3 BRs (i.e., hyper-expanded capsids), in-gel propagation increases production of α-sheet-rich herpes virus assemblies. In support, correlation of herpes virus-1 (HSV-1) infection with Alzheimer disease increases for individuals with the APOE-e4 allele (lipoprotein precursor gene) [48]. This genotype is also associated with changes in the brain microvascular basement membrane, which is basically a gel [49]. The hypothesis is that the latter changes promote formation of Alzheimer disease-triggering BR analogues during HSV-1 infection of the basement membrane.

If the above hypothesis is correct, a corollary is that therapeutic viruses can be developed for neurodegenerative diseases. Specifically, therapeutic viral α-sheet would detoxify amyloid protein α-sheet by co-assembly, followed by one or both of the following: (1) speeding conversion to β-sheet, (2) destabilizing or detoxifying toxic α-sheet (additional details [47]).

## 4. Materials and Methods

### 4.1. Propagation of Phages: Termination, Fixation and Agarose-Embedding of Liquid Cultures

Phage T3 plaques were produced by traditional means [50] in a Petri plate. The temperature was 30 °C unless otherwise indicated. The lower gel was 1.0% agar in 1.0% Bacto tryptone and 0.11 M NaCl. The upper, plaque-supporting gel was 0.60% agarose (Seakem Gold; Lonza, lot 50150, Basel, Switzerland) in 2×LB medium: 2.0% Bacto tryptone, 1.0% Bacto yeast in 0.1 M NaCl. The host was *Escherichia coli* BB/1. Host cells added to the upper layer, molten gel had been propagated in aerated liquid culture overnight at 30 °C in 2×LB medium.

Liquid culture in 2×LB medium was started with 1:200 dilution of an overnight *E. coli* culture in 2xLB medium. Incubation was then performed at 30 °C with aeration. The cells were infected with T3 phage at a multiplicity of 5 when a concentration of 4 × 10^8^ cells per ml was reached, as determined by counting in a Petroff-Hausser counting chamber. The phage infection (burst size at spontaneous lysis = 50–100) was continued until terminated by dilution of 3.0 mL of infected cells into 27 mL of the following fixative and vortexing: 4.0% formaldehyde, 1.0% glutaraldehyde, 0.11 M sodium phosphate, pH 7.3. The times of termination are indicated in the Results Section. Fixation was continued for 1.0 h at room temperature.

The fixed, infected bacteria were pelleted at 30 °C, 8000 rpm for 5 min in a Beckman JA25.5 rotor used in a Beckman Avanti J-25 centrifuge. The pellet was resuspended in 50 μL 2×LB medium and equilibrated at 37 °C. Then, 50 μL of the following was added and mixed: 1.2% agarose (Seakem Gold) in 2×LB medium equilibrated at 55 °C. This mixture was placed in a microtiter plate and gelled by incubation for 10 min on ice. The gelled mixture was then processed by the procedure used for plaques in the next section.

To produce plaques in the presence of proflavine, proflavine was added to the top and bottom layer gels in a Petri plate (20 μg/mL final concentration). Incubation was performed as done without proflavine, at temperature indicated in the Results Section.

### 4.2. Thin Sectioning and Electron Microscopy (EM-TS)

To prepare plaque contents for EM-TS, a 2–4 mm segment of plaque-supporting agarose gel was excised with a sterile, thin spatula and fixed by submersion in the fixative used in the previous section. The gel segment encompassed the border between clear interior and turbid edge of the plaque. After fixation for at least 2.0 hr at room temperature, the gel was (1) washed for 5 min in 1.5 mL of 0.1 M sodium phosphate, pH 7.3 and (2) post-fixed for 30 min, at room temperature, in 1.0% osmium tetroxide dissolved in Zetterqvist’s buffer [51].

A fixed, plaque-supporting agarose gel was then dehydrated in ethylene/propylene oxide and embedded in Epon 812 resin by use of procedures previously described [16]. The resin was polymerized at 85 °C overnight, in a flat-embedding BEEM capsule. An embedded gel was thin-sectioned with a Leica EM UC6 microtome and diamond knife. Thin sections were cut, floated and adhered to a 150-mesh grid, as previously described [16]. The sections used were color-selected [52] for ~100 nm thickness. Finally, sections were stained by one of the following procedures, as indicated in the Results, Section 2. (1) Unless otherwise stated, the sections were stained with 7% uranyl acetate for 30 s, followed by Reynold’s lead citrate [53] for 20 s both in a microwave oven (0.035 W cm^−3^). Procedure of this type generates relatively heavy staining of the packaged DNA of phages [17,18,19,20,21]. (2) The above staining was either replaced by or followed by the staining procedure in the next paragraph.

First, a section was stained with 5% phosphotungstic acid (PTA), adjusted to a pH of 3.2 with NaOH. The staining was performed for 20 s in a microwave oven (0.035 W cm^−3^). Then, the section was washed with 3 drops of 0.02 M acetic acid that had been adjusted to a pH of 3.0 with sodium hydroxide. The inspiration for developing this acidic PTA (A-PTA) staining was the previous success of an A-PTA precipitate staining used pre-dehydration [28]. The low pH of the wash suppressed movement of PTA precipitate [28].

Electron microscopy was performed with a JEOL JEM-1400 electron microscope. Images were recorded with an AMT image capture engine Version: 7.

### 4.3. Interpretation of Electron Micrographs

If a capsid’s shell is resolved during observation of a thin section, then (1) an equator of the capsid’s shell is in the section and (2) the observed diameter of the capsid does not have to be corrected for the position of the section’s surfaces. This conclusion is based on analysis of serial sections of phage T4 capsids [21]. T4 phages and capsids of this previous study are each expected to be intersected by a section with a probability higher than it is for the smaller T3 versions observed here.

Furthermore, if an isometric particle is made visibly asymmetric (ellipsoidal) by the process of specimen preparation, the cause is compression during thin sectioning. The length of the long axis of the ellipse is the most accurate rendition of the diameter of the particle before compression [21]. This conclusion is assumed here during measurements of particle diameters.

## Supplementary Materials

The following are available online at https://www.mdpi.com/1424-8247/13/1/18/s1. Figure S1: Unsegmented version of Figure 1, an image obtained by EM-TS of a phage T3 plaque.
