# Supplementary figures and images for: Electron Microscopy of In-Plaque Phage T3 Assembly: Proposed Analogs of Neurodegenerative Disease Triggers
Source: Pharmaceuticals (Basel). 2020 Jan 18;13(1):18. doi: 10.3390/ph13010018 (PMC7170049; doi:10.3390/ph13010018)

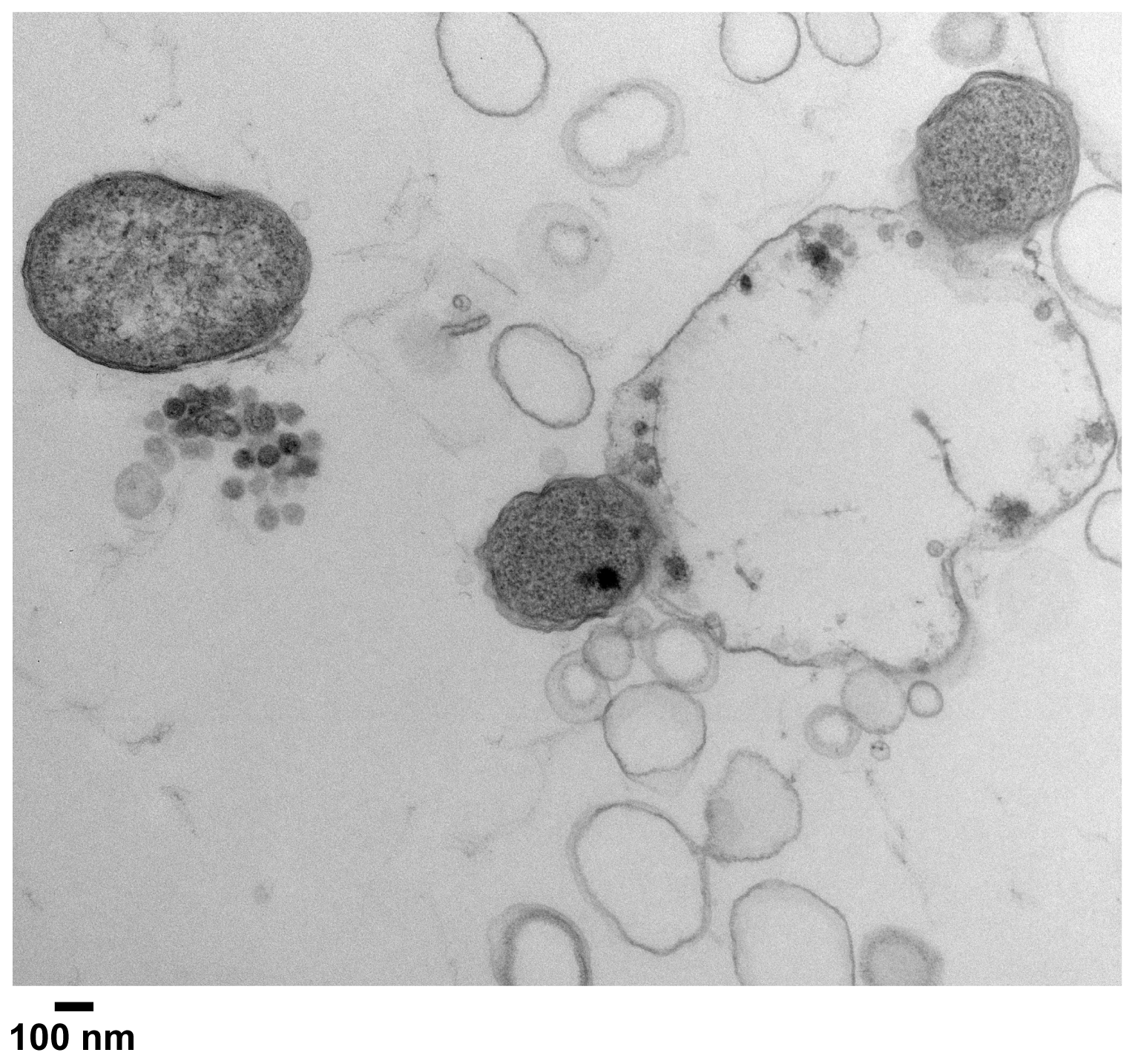

Supplement: Supplementary file 1 [file pharmaceuticals-13-00018-s001.zip › pharmaceuticals-686941-supplementary.tif]
